# COVID-19 in the Greater Mekong Subregion: how resilient are rural households?

**DOI:** 10.1007/s12571-020-01069-0

**Published:** 2020-07-13

**Authors:** Hermann Waibel, Ulrike Grote, Shi Min, Trung Thanh Nguyen, Suwanna Praneetvatakul

**Affiliations:** 1 Institute of Development and Agricultural Economics, Leibniz Universität Hannover, Königsworther Platz 1, 30167 Hannover, Germany; 2 Institute for Environmental Economics and World Trade, Leibniz Universität Hannover, Königsworther Platz 1, 30167 Hannover, Germany; 3 College of Economics and Management, Huazhong Agricultural University, No.1 Shizi Shan Street, Hongshan District, Wuhan, 430070 China; 4 Department of Agricultural and Resource Economics, Kasetsart University, 50 Ngam Wong Wan Rd, Ladyaow Chatuchak, Bangkok, 10900 Thailand

**Keywords:** COVID-19, Greater Mekong Subregion, Resilience, Rural households

## Abstract

In this paper we submit some thoughts on the possible implications of the COVID-19 pandemic for rural people in the countries of the Greater Mekong Subregion (GMS). We base our observations and conclusions on our long-term research experience in the region. The paper focuses on the economics of rural households during this crisis period and its aftermath. We conclude that country differences clearly exist due to their different stages of development. However, while rural households belong to the Corona risk groups, they are also resilient to such a shock. We submit that Governments in the GMS should strengthen policies that conserve the safety-net function of rural villages.

The Coronavirus pandemic has significant macroeconomic implications, which may vary in intensity among the countries of the Greater Mekong Subregion (GMS) due to differences in the resilience of their economies and the capacities of their public health systems. Based on the John Hopkins University reports, so far, in terms of confirmed Corona infections the ranking is China, followed by Thailand, Vietnam, Cambodia and Laos is lowest. We underline that rural areas in the GMS are of significant importance for their respective countries. Farmers supply food to domestic markets and produce agricultural commodities like rice, rubber, fruits and vegetables for export. However, even before Corona, the region was affected by dramatic environmental problems as a result of economic development in the area of the Mekong river and its tributaries.

This paper briefly investigates the expected implications of COVID-19 for the rural areas of the GMS countries.

We start our brief review of the GMS countries with China. We focus on Xishuangbanna (XSBN) in Southwest China as an example. In this region, almost 80% of the land has been used to plant natural rubber (Min et al. 2017). In the non-farm sector, tourism has become a pillar industry. Before the Corona pandemic, farmers were already hit hard by declining rubber prices. The sudden outbreak of the Coronavirus has affected the entire economy in China and in XSBN, especially the tourism industry. Hence, off-farm employment opportunities as a major coping strategy for declining income from rubber were suddenly blown away. Intensifying home gardening and planting food crops have become an option for some farmers in the medium run. In the short run, increasing natural resource extractions are possible but yet limited because resource stocks have been depleted as a result of the dramatic changes of land use in the past. Economic recovery, which has already started in China, will come with some delay to the rubber farmers in XSBN, largely depending on the recovery time of the automotive industry.

In Laos, infections are among the lowest in the world. If this does not change radically, the effect of COVID-19 on human life and the rural economy will be small. In the western part of the country, the Lao economy has close connections to Thailand. According to data from the Thai Ministry of Labour some 170 000 migrant workers are in Thailand, i.e. less than 5% of Laos’ labour force of 3.6 million (Australian Aid 2019). Thus, lock-down of the Thai economy will have some negative impact on Laos. On the other hand, overall, agriculture in Laos is still subsistence-orientated and natural resource extraction is a major source of household wealth (Parvathi and Nguyen 2018). However, rural households respond, especially to health shocks, with intensifying natural resource extraction (Nguyen et al. 2018). Hence, the Corona crisis is likely to augment the ongoing natural resource depletion in Laos.

A similar conclusion can be drawn for Cambodia, where the non-farm sector has been growing rapidly in the past. Although the number of migrant workers in Thailand with some 390 000 (IOM 2019) is higher than in Laos this also corresponds to about 5% of the total labour force. On the other hand, the rate of natural resource extraction is even beyond that of Laos. In addition to deforestation, Cambodia’s fish resources are dwindling mainly due to hydropower development upstream. Furthermore, poorer households are heavily dependent on fishing as a source of income and food (Hartje et al. 2018). Our research showed a strong resilience of rural households to external shocks primarily due to well-functioning community and family safety nets (Nguyen et al. 2020). A pandemic like COVID-19 can significantly weaken these networks, for example as a result of job loss in the non-farm sector.

In Vietnam, driven by high input intensity, especially in the high potential agricultural areas (e.g. Red River and Mekong Deltas), outstanding levels of productivity have been achieved though unsustainable agricultural practices and climate change have already started to weaken land use systems. Nevertheless, resilience of rural households may still be strong, at least in the short to medium term. However, farms are small which makes them dependent on external inputs. Although Vietnam’s “Corona policy” has been to keep strategically important industries in operation, agricultural input supply chains are weakened. In addition, remittances from household members working in urban areas which have been widely used to finance farm inputs (Nguyen et al. 2019) are now missing as many migrant workers were laid off. It is therefore quite likely that agricultural growth in Vietnam will slow down. In the upland and mountainous areas of Vietnam, the impact of the COVID-19 pandemic may be similar to Cambodia and Laos with an increased engagement of households in natural resource extraction, especially logging (Völker and Waibel 2010).

Finally, in Thailand, the economy has been hit hard by an almost complete lock-down in the Greater Bangkok area starting in mid-March. One of the effects was the return migration of millions of workers to their natal villages, mostly to Northeast Thailand. This has increased the risk of infections in the rural areas where initial infection rates were very low. At the same time, health infrastructure and social protection is particularly weak in rural areas. However, generally, resilience of Thai agriculture is strong. Although yields are lower (as compared to Vietnam), farm sizes are larger, ownership of assets is high and rural microfinance is widely available. In addition, many rural people in Thailand have embraced the self-sufficiency economy concept promoted by their past King Bumiphol (Mongsawad 2010). A spirit of social adaptation, social support based on local safety nets and public consciousness of political, economic, environmental and health crises have generated a wide array of coping strategies that can be helpful to soften problems in connection with the Corona crisis. Conversely, financial problems due to high levels of indebtedness of rural households (Chichaibelu and Waibel 2018) could be aggravated further by the health crisis.

This short review of the conditions in these five countries in the GMS enables us to draw some general lessons. First, we can say that rural people in the GMS countries belong to the risk groups of COVID-19, primarily because of low levels of health protection. Public health services in rural areas are much weaker than in the capital and other major cities. Second, rural households in the five countries have become dependent on off-farm income. For many rural households, income from agriculture before COVID-19 has been less than half of annual household income. Industry lock-downs have wiped out remittances as a major income source. More so, especially in Thailand, it has driven millions of migrant workers back to their rural villages. Third, and as a consequence of the swift return migration, demand for food in rural households will increase and, at least in the short run, this will increase pressure on the already stressed natural resources. More people will ignore the barely enforceable access rules and more people will extract more natural resources to pay for food and where ever possible, collect and hunt for food. Fourth, in terms of agricultural output, the lock-down may interrupt supply chains on both, the input and the output side and hence food prices may increase. As a result, farmers may adopt de-intensification strategies which would reduce aggregate agricultural output.

Fifth, on the plus side, rural households in GMS countries are resilient. They have multiple sources of income including own crop production, home gardening, raising livestock and homestead aquaculture as well as income from logging, hunting, fishing and other collections in open access areas. In the medium run they can also diversify their crop and livestock portfolio. In several ways, rural households become the safety net for the laid-off labour force in urban areas. An anecdote from Vietnam underlines this. A migrant worker from the Mekong Delta working in the service industry in Hanoi who was not able to return home before travel restrictions were imposed, said: “*before, I regularly sent money to my mother in my village in Can Thoe Province but now I am begging her to send money to me*”. This illustrates that rural safety nets can be considered as a national resource with a high option value. The COVID-19 case underlines that emerging market economies should implement policies that help to maintain this “resource”, because they may still need it in times of future crises.

Finally, another reason why we argue that resilience of rural households in the GMS is high, is their long lasting experience with shocks. In numerous household surveys conducted by the authors, when asking respondents about past shocks, quite often households reported shocks but did not rate them as serious, simply because they had it so many times. The imperfect and dysfunctional insurance markets in rural areas have made them innovative and adaptive to cope with shocks and to some extent also resistant to them. A good example of this is the case of the Tungro virus, one of the most destructive diseases of rice. Asian farmers sometimes call it the “cancer of rice”. The Tungro disease in rice has some similarities with Coronavirus in humans. It cannot be detected immediately and there is no direct method of control. The only effective measure is what plant pathologists call “integrated pest management”, with resistant varieties, strengthening plant health, asynchronous planting and irrigation management and pest surveillance, as its central components. Tungro is an example how farmers have learned to live with a serious disease and continue to grow rice successfully.

Governments in the GMS countries have implemented policies aimed to help rural households with the pandemic. In China (XSBN), the Government has provided rice to poor smallholder rubber farmers and implemented price control schemes as well as promoted rural E-commerce and allowed the establishment of roadside booths to strengthen direct marketing of farm produce. In Vietnam, the government implemented cash transfers of one million VND/household/month (US$43) for the months of April to June for poor and near-poor households, for workers who lost their job and small-scale businesses. Similarly, in Thailand, the government implemented cash transfer programs for people who lost their job as well as financial support for rice farmers of 1000 THB per rai (about US$200/ha) and loan restructuring measures to ease the pressure of households, already debt-stricken before the crisis. Cambodia implemented minor support measures in the garment sector (20% of minimum wage) and both Cambodia and Laos implemented COVID-19 response projects in the health sector financed through support from the World Bank.

While the effectiveness of these immediate response measures is yet unknown and can only be assessed after rigorous impact studies have been conducted, clearly, COVID-19 increases the challenge for governments in the GMS countries to draw the right lessons from this crisis. We submit that this is a good point in time to re-assess the policies of the past and develop a coherent set of rural development policy measures that recognize the multi-functionality of agriculture and the role of rural villages in future development. Modern natural resource management technologies and rural based Small and Medium Enterprises (SMEs) making effective use of the advancement in information and communication technology are major factors. Besides, significantly strengthening health systems in rural areas, including, for example, significant improvements in the sanitary conditions will help to maintain and strengthen rural areas’ resilience to the crises to come.
